# West Australian parents’ views on vaccinating their children against COVID-19: a qualitative study

**DOI:** 10.1186/s12889-023-16645-0

**Published:** 2023-09-11

**Authors:** Samantha J. Carlson, Katie Attwell, Leah Roberts, Catherine Hughes, Christopher C. Blyth

**Affiliations:** 1 Wesfarmers Centre of Vaccines and Infectious Diseases, Telethon Kids Institute, Perth, WA Australia; 2 School of Social Sciences, The University of Western Australia, Perth, WA Australia; 3 Immunisation Foundation of Australia, Perth, WA Australia; 4 School of Medicine, University of Western Australia, Perth, WA Australia; 5 Department of Infectious Diseases, Perth Children’s Hospital, Perth, WA Australia; 6 Department of Microbiology, PathWestLaboratory Medicine, Perth, WA Australia

**Keywords:** COVID-19, Children, Adolescents, Vaccination, Health decision-making

## Abstract

**Background:**

Australian children and adolescents were among the last local cohorts offered COVID-19 vaccines. Despite promising initial uptake, coverage subsequently plateaued, requiring further efforts to improve access and build parents’ recognition of the importance of COVID-19 vaccination. We sought to understand West Australian (WA) parents’ willingness to vaccinate their children to inform strategies for improving uptake at the time in which they were becoming eligible.

**Methods:**

We undertook in-depth qualitative interviews with 30 parents of children aged 5–17 years from June – December 2021. During this period, children aged 12–15 years became eligible for vaccination; children aged 5–11 years became eligible shortly thereafter. Data were thematically analysed in NVivo.

**Results:**

Most parents intended on vaccinating their children once eligible. Parents sought to protect their children, to protect the community, to resume travel, and to get back to “normal”. They reflected that vaccination against key infectious threats is a routine activity in childhood. Some were concerned about the vaccine, particularly mRNA vaccines, being new technology or impacting fertility. “Wait-awhiles” wanted to see what other parents would do or were delaying until they felt that there was a higher risk of COVID-19 in WA. Most parents of younger children wanted their child to be vaccinated at the general practice clinic due to familiarity and convenience. Parents were particularly eager for clear and consistent messaging about vaccination of children and adolescents, including safety, importance, scientific evidence, and personal stories.

**Conclusion:**

For future pandemic vaccinations pertaining to children, governments and health officials need to address parents’ concerns and meet their preferences for the delivery of the vaccine program to children and adolescents.

## Background

In 2021, approximately 25% of total Coronavirus Disease 2019 (COVID-19) cases in Australia were in children aged under 16 years [1]. Severe outcomes in this age group were infrequent with less than 3% of confirmed cases hospitalised [1]. However, children with underlying health conditions including asthma, obesity, prematurity and immunocompromising conditions are at greater risk of severe COVID-19 [2–4]. Although uncommon, some children experience persistent symptoms following initial infection, [5] and approximately 1 in 2,500 develop Multisystem Inflammatory Disease in Children (MIS-C)/Paediatric Inflammatory Multisystem Syndrome (PIMS-TS) [4]. In addition to the health impacts of COVID-19, there are also social, emotional, financial and educational impacts of infection and isolation periods [6, 7]. Vaccination has the potential to mitigate against these harms and as such is recommended for all children from 5 years in Australia, and more recently for young children with specific medical risk factors [8–10].

In Australia, adolescents aged 16 years and older were the first non-adult population to be offered the vaccine, though they were at the bottom of the overall vaccine rollout priority list [11] due to their lower risk from the disease compared to older populations. In Western Australia (WA), 16- and 17-year olds were first offered the vaccine from mid-August 2021, [12] approximately six months into the national rollout. For children aged 12 years and older, the Pfizer vaccine was registered for use in July 2021, and Moderna in September 2021; [11] children from 12 years in WA were offered either vaccine from mid-September 2021 [13]. For children aged 5 – 11 years, the Pfizer vaccine was registered for use in December 2021; [11] parents in WA could then vaccinate their children in this age group from mid-January 2022 [12]. Finally, for children aged 6–11 years, the Moderna vaccine was registered for use in February 2022 and available shortly thereafter. As of August 2022 in WA, first dose uptake in adolescents aged 12–15 years was 86% with 80% overall having received two doses, and in children aged 5–11 years, first dose uptake was 57% with 43% overall having received two doses [14].

Social scientists working in many countries and social contexts have extensively researched parents’ attitudes towards vaccination against established childhood diseases. Many studies investigate vaccine hesitancy and the reasons why parents doubt or refuse vaccines, which include concerns about ingredients and side effects, beliefs that too many concurrent vaccines are given, adherence to natural or alternative lifestyles, beliefs that children are either too healthy or too vulnerable to be vaccinated, and distrust in health and medical authorities [15–21]. However, other studies investigate what motivates parents to vaccinate their children, often in the face of exposure to vaccine scares or misinformation [22, 23].

Scholars globally have conducted a number of studies regarding parents’ attitudes and intentions to vaccinate their children against COVID-19. A qualitative study conducted in the United Kingdom found almost half of parents and guardians would vaccinate their child/ren for COVID-19, with those of English and Irish ethnicity more likely accept the vaccine [24]. Another qualitative study in the United States found that COVID-19 vaccine hesitancy was not limited to parents who had previously delayed, refused or hesitated about childhood vaccines, and that parents who fully vaccinated for other diseases were influenced by factors including information sources, social networks and parents’ own pandemic experiences [25]. Further studies conducted globally reported mixed attitudes towards paediatric COVID-19 vaccination; parents were more likely to vaccinate their children if they were willing to vaccinate themselves, [26, 27] if they believed social distancing was worthwhile, [28] if their child was up to date with childhood vaccinations, [29] and if they planned to vaccinate their child against influenza [30, 31]. Again, participants’ experience of the pandemic were important, as was their desire to reduce the spread of COVID-19 [32].

There has been less work conducted in Australia. In early 2021, ahead of COVID-19 vaccines being available in Australia, 48% of ~ 1,000 parents surveyed nationally said they intended to vaccinate their children and adolescents against COVID-19 once they were able to, 38% were unsure, and 14% would not [33]. Trust in doctors was found to be a predictor of acceptance, and open-ended questions revealed parents were worried that for children, the risks of vaccination outweighed the benefits. What remained unknown, and thus was our aim to understand, is what West Australian parents intended to do for their children once a COVID-19 vaccine became available for them. Understanding their intent was important because, at the time of children becoming eligible, as a results of border closures and other non-pharmaceutical interventions, Western Australia had very little COVID-19 disease and no community transmission (total cases from February 2020 to December 2021 was only 1,185) [34].

## Methods

This study formed part of the larger Coronavax project. Coronavax: Preparing Community and Government is an interdisciplinary project focused on understanding community perspectives towards COVID-19 vaccinations in WA via in-depth interviews conducted with specific population groups (such as parents), as well as studies of social media and policymakers [35]. Ethics for the project was obtained from CAHS HREC RGS000000445.

### Participant recruitment

Participants signed up via the online survey portal REDCap [36, 37] which was advertised through traditional and social media promotion (with the aid of our consumer representative). Participants were asked a range of demographic questions, including whether or not they care for children under the age of 18 years. Any with children aged 5 – 18 years were then invited for an interview and were either emailed or called to be invited to participate in an in-depth interview. Given the way in which participants were recruited, the interviewers had no prior relationship with the participants.

### Data collection

Participants were interviewed via phone, teleconferencing software or face-to-face by SJC (an experienced qualitative researcher) and LR (a junior qualitative researcher guided by SJC) between June – December 2021. The mode of interview was dictated by the parent, with most choosing a phone interview.

The Coronavax interview schedule was developed by a multi-disciplinary team; the additional “parent” module utilised here was informed by the team’s prior expertise in childhood vaccination attitudes and uptake and co-designed with an experienced community representative (author CH). Throughout the approximately 1-h interview, parents were asked their intentions to vaccinate their children, their reasons for this intention, how they came to their decision, their preferences for where they’d like to take their children for vaccines, their level of concern regarding COVID-19, what their social network was doing and saying, and their COVID-19 vaccine information needs. Parents were also asked their views about mandating COVID-19 vaccinations for children. All interviews were audio recorded and transcribed verbatim. Transcripts were not returned to participants were comment.

Demographic data collected in the REDCap survey included COVID-19 vaccine status, gender, level of education, country of birth, language spoken at home, postcode, employment industry, age of children, and vaccine status of children. The REDCap survey was only provided in English. Further, those who signed up self-selected “yes” or “no” in REDCap to whether their child had a ‘severe health condition that increased the risk of COVID-19 severity.’ They were asked to elaborate on the condition during the interview, and then their answers reviewed by a clinical expert (author CCB).

### Data analysis

**Fig. 1:**
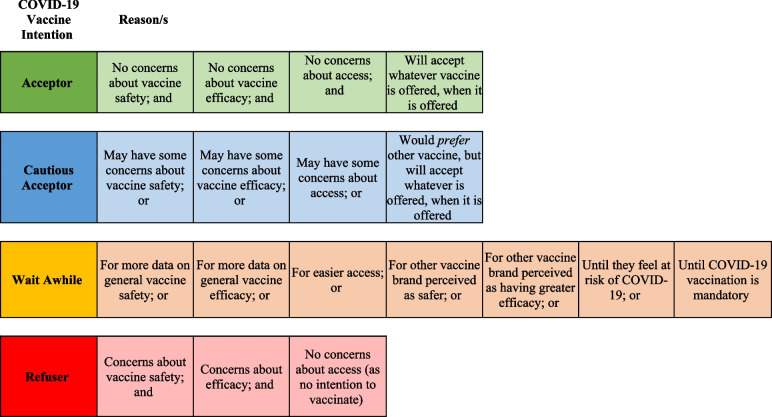
COVID-19 vaccine intentions model. Note: Figure from Carlson SJ, McKenzie L, Roberts L, Blyth CC, Attwell K. Does a major change to a COVID-19 vaccine program alter vaccine intention? A qualitative investigation. Vaccine. 2022;40(4):594–600. Published under a Creative Commons Attribution-NonCommercial-No Derivatives License

Interviews were conducted until we reached data saturation: we estimated this would be between 20 and 30 interviews. Data collected during the interviews were thematically analysed using inductive methods by SJC in NVivo 20. Following this inductive analysis, SJC then employed deductive analysis to classify parents’ intention to vaccinate their child(ren) as per the team’s COVID-19 vaccine intentions model developed for a prior study (Fig. 1) [38]. This model divides intentions into four categories: 1) acceptor, 2) cautious acceptor, 3) wait awhile, and 4) refuser. All authors gave feedback on the themes and coding that SJC described and categorised before SJC finalised the analysis. Pseudonyms are applied to all parents and children. Participants did not provide feedback on the findings. Any data on mandates was not used in this analysis and instead merged with data collected from parents of children aged less than five years for a separate analysis [39].

## Results

We interviewed 30 parents for this study. Of the 30 interviews, 16 (53%) were conducted by LR, and 14 (47%) by SJC. Interview duration ranged between 34 and 107 min, with an average of 65 min.

Of the 30 participants, 66% had received at least one COVID-19 vaccine dose themselves at the time of interview, 73% were female, 73% had a university degree, 60% were born in Australia, 97% spoke English at home, 47% identified as Christian, 87% lived in the Perth metropolitan region, and 53% resided in the most advantaged postcodes in WA. The most common industries participants worked in were education and training (17%), and mining (17%), followed by health care and social assistance (13%) and public administration and safety (13%) (Table 1).

**Table 1:** Demographics of 30 West Australian parents of children aged 5 – 17 years

Characteristic (parent)	Number (%)
*Received at least one COVID-19 vaccine dose*	
Yes	20 (66)
No	10 (33)
*Age group (years)*	
30 – 39	3 (10)
40 – 49	17 (57)
50 – 59	10 (33)
*Gender*	
Female	22 (73)
Male	8 (27)
*Highest level of education*	
Year 12 or equivalent	5 (17)
TAFE/apprenticeship or equivalent	3 (10)
University degree	22 (73)
*Number of children aged* < *18 years* ^a^	
1	9 (30)
2	15 (50)
3	6 (20)
*Born in Australia*	18 (60)
*English spoken at home*	29 (97)
*Lives in Perth metropolitan region*	26 (87)
*Employment industry*	
Construction	3 (10)
Education and Training	5 (17)
Financial and Insurance Services	1 (3)
Health Care and Social Assistance	4 (13)
Home duties	2 (7)
Manufacturing	3 (10)
Mining	5 (17)
Professional, Scientific, and Technical Services	3 (10)
Public Administration and Safety	4 (13)
*SEIFA decile score* ^b^	
1 – 4	5 (17)
5 – 7	9 (30)
8 – 10	16 (53)

^a^Some parents also had children aged 18 + years – these adult children are not included in sum

^b^The SEIFA (Socio-Economic Index For Areas) is a ranking system developed by the Australian Bureau of Statistics. It “ranks areas in Australia according to relative socio-economic advantage and disadvantage” based on information gathered from the Census. This data was developed from the “Ranking within State or Territory (Western Australia)” > Decile numbers from the 2016 “Postal Area (POA) Index of Relative Socio-economic Advantage and Disadvantage.” [40]. The higher the number, the more well-off the area is, based on postcodes

The 30 parents represented 57 children aged 5–17 years: 51% (*n* = 29) were aged 5–11 years, and 49% (*n* = 28) were aged 12–17 years. Of all children, one-third (*n* = 19, 33%) were eligible to be vaccinated at the time of interview, with 47% (9/19) already vaccinated. All parents self-reported that their children were up-to-date with routine vaccines, except for one child who was not fully vaccinated per the advice of their medical specialist. Of those for which we have the data, 59% (13/22) of the children aged 5–11 years, and 33% (6/18) of the children aged 12–17 years, usually received an influenza vaccine (Tables 2 and 3).

**Table 2:** Vaccine intentions and behaviour of parents of children aged 5 – 11 years

Parent’s details				Child’s details			
**Pseudonym**	**Parent’s COVID-19 vaccine intention for self**	**Parent vaccinated**	**Parent’s COVID-19 vaccine intention for child**	**Age (years)**	**Comorbidity that increased risk of severe COVID-19 infection**	**Child up-to-date with routine vaccines?**	**Child usually receives annual influenza vaccine?**
Brooke	Accept	Yes	Accept	11	Yes	Yes	Yes
Nikki	Accept	Yes	Accept	6	No	Yes	Yes
				9	No	Yes	Yes
Xena	Accept	Yes	Accept	10	No	Yes	Yes
Rosemary	Accept	Yes	Accept	11	No^b^	Yes	Yes
Jim	Accept	Yes	Accept	10	No	Yes	Yes
				10	No	Yes	Yes
				11	No	Yes	Yes
Samuel	Accept	Yes	Accept	10	No	Yes	Unsure^a^
Louise	Accept	No	Accept	6	No	Yes	Unsure
				7	No	Yes	Unsure
Cindy	Cautiously Accept	Yes	Accept	7	No	Yes	No
				11	No	Yes	No
Marty	Cautiously Accept	Yes	Accept	8	No	Yes	No
				10	No	Yes	No
Adele	Wait awhile	No	Accept	8	No	Yes	Yes
Nicholas	Accept	Yes	Cautiously accept	8	No	Yes	Unsure
				10	No	Yes	Unsure
Tracey	Cautiously Accept	Yes	Wait awhile	5	No	Yes	Yes
				9	No	Yes	Yes
Jessica	Wait awhile	No	Wait awhile	7	No	Yes	Unsure
				9	No	Yes	Unsure
Claire	Wait awhile	No	Wait awhile	8	No	Yes	Yes
				10	No	Yes	Yes
Anne	Refuse	No	Refuse	5	No	Yes	No
				5	No	Yes	No
Eden	Refuse	No	Refuse	6	No	Yes	No
				8	No	Yes	No
				9	No^b^	Yes	No

^a^Was not discussed during interview, so research team unsure if child usually receives annual influenza vaccine

^b^Parent indicated in demographic survey that child did have a comorbidity that increased the risk of a severe COVID-19 infection. A clinical review of the comorbidities indicated that the child was not at increased risk and thus is recorded here as not having a comorbidity

**Table 3:** Vaccine intentions and behaviour of parents of children aged 12 – 17 years

Parent’s details				Child’s details					
**Pseudonym**	**Parent’s COVID-19 vaccine intention for self**	**Parent vaccinated**	**Parent’s COVID-19 vaccine intention for child**	**Age (years)**	**Comorbidity that increased risk of severe COVID-19 infection**	**COVID-19 vaccine available for age at time of interview?**	**Child vaccinated against COVID-19?**	**Child up-to-date with routine vaccines?**	**Child usually receives annual influenza vaccine?**
Jurgen	Accept	Yes	Accept	13	Yes	Yes	Yes	Yes	No
Michelle	Accept	Yes	Accept	13	No	Yes	Yes	Yes	Unsure^a^
				17	Yes	Yes	Yes	Yes	Unsure
Xena	Accept	Yes	Accept	12	No	Yes	Yes	Yes	Yes
Brooke	Accept	Yes	Accept	14	No	Yes	Yes	Yes	Yes
Pippin	Accept	Yes	Accept	15	No	Yes	Yes	Yes	No
Sheena	Accept	Yes	Accept	17	No	Yes	Yes	Yes	No
Alice	Accept	Yes	Accept	17	No	Yes	No	Yes	Yes
Sal	Accept	Yes	Accept	15	No	Yes	No	Yes	No
Diana	Accept	Yes	Accept	16	No	Yes	No	Yes	No
Samuel	Accept	Yes	Accept	13	No	Yes	No	Yes	Unsure
Rosemary	Accept	Yes	Accept	14	No	No	N/A	Yes	Yes
				16	No^b^	No	N/A	Yes	Yes
Louise	Accept	No	Accept	12	Yes	No	N/A	No	Unsure
Cindy	Cautiously Accept	Yes	Accept	14	No	Yes	Yes	Yes	No
Adele	Wait awhile	No	Accept	13	No	No	N/A	Yes	Yes
Caroline	Cautiously Accept	No	Cautiously Accept	15	No	Yes	No	Yes	Unsure
Milly	Cautiously Accept	Yes	Cautiously Accept	16	No	Yes	Yes	Yes	No
Sebastian	Accept	Yes	Wait awhile	15	Yes	No	N/A	Yes	No
				16	No	No	N/A	Yes	No
Julia	Cautiously Accept	Yes	Wait awhile	16	No	Yes	No	Yes	Unsure
				17	No	Yes	Yes	Yes	Unsure
Jessica	Wait awhile	No	Wait awhile	12	No	No	N/A	Yes	Unsure
Messi	Wait awhile	No	Wait awhile	15	No^b^	Yes	No	Yes	Unsure
				16	No	Yes	No	Yes	Unsure
Ruby	Refuse	No	Refuse	14	No^b^	No	N/A	No	No
				17	No	Yes	No	No	No
Lisa	Refuse	No	Refuse	16	No	Yes	No	Yes	No

N/A: child unable to be vaccinated despite parent’s intention as vaccine not available at time of interview

^a^Was not discussed during interview, so research team unsure if child usually receives annual influenza vaccine

^b^Parent indicated in demographic survey that child did have a comorbidity that increased the risk of a severe COVID-19 infection. A clinical review of the comorbidities indicated that the child was not at increased risk and thus is recorded here as not having a comorbidity

Five children had comorbidities increasing the risk of severe COVID-19, including asthma, congenital heart disease, immunoglobulin (IgG) deficiency, type 1 diabetes and prematurity (born at 35 weeks). A further four parents believed that their children had such a comorbidity, but clinical review by CCB determined that the comorbidities were not active or did not increase the risk of severe COVID-19 (e.g. behavioural concerns, learning difficulties, allergies, genetic conditions).

### COVID-19 vaccine intentions for child/ren

Despite nearly all children being up-to-date with their routine vaccines (Tables 2 and 3), parents’ intentions to vaccinate them against COVID-19 varied. Most intended to, and many of these parents had themselves been vaccinated (Tables 2 and 3). The most common reason to vaccinate was to protect the child from COVID-19. Other reasons included trusting authorities’ recommendations, getting back to “normal,” for “herd immunity” and to protect other more vulnerable individuals, to travel, and to prevent long COVID.

First and foremost, it would be about [protecting the children]. Secondly, it would be about [protecting] my parents…but then the wider community. It’s protecting everyone – Rosemary

I think once we're all fully vaccinated, I will feel a big sense of relief. That means we've done enough to protect ourselves from COVID, and then hopefully the flow-on effects in protecting…the wider family and friends – Xena

Some parents were ‘cautious acceptors’. Caroline, who was taking her 15-year-old son to get vaccinated after the interview, had some concerns about short-term side effects impacting his quality of life:

*We actually wanted to get him vaccinated before we sent him back to boarding school, but he had a footy [football] carnival and I just didn't want him to have a reaction … ‘cause he's had a broken finger for the last three months, so this was, like, the one time he could go out and do something, and I was like, “Oh, do I risk it?”* – Caroline

Nicholas, who was “*99% sure*” he’d vaccinate his children, reported “*slight hesitation*” because of the vaccine being new. Milly, who was from and worked within a multicultural community in Perth, initially had concerns for both herself and her 16-year-old daughter. Milly’s concerns derived from rumours within her community about vaccine safety but also theories about the vaccine becoming a tracking device:

*If you can't read and write in English, it’s not your language and you don't know what's going on and anyone coming and saying, “Oh, it's not a good thing, don't get it done,” you’re just gonna have to listen to them….At first it was like, “Oh, you’re gonna be followed, you’re gonna be followed, they’ll know your whereabouts and everything.” [And so I’m thinking] “Oh, I’m not gonna get it done, why would I get it done?” Like they’re gonna put something in your body and it's not really safe. And so I was like everyone else, I was like, “I'm not getting it done.”* – Milly

Both mother and daughter ultimately got vaccinated.

Some parents were also intending to ‘wait awhile.’ An interesting example is Julia. Her 17-year-old son, Jackson, got vaccinated “*without even consulting us, him and his girlfriend went and had the first jab*.” Julia had wanted Jackson to wait until his parents had been vaccinated and recovered from potential side-effects. For her 16-year-old son, Jonathan, Julia felt at the time of interview that there was little risk from COVID-19. Consequently, they were prioritising other life decisions, such as selecting subjects for the final year of school, before having a conversation about the vaccine.

Sebastian, a vaccinated father, was waiting a while to vaccinate his children due to his wife’s concern about “*the speed in which it’s been introduced.”* Other parents were waiting for more information on safety. This was particularly true for parents of children aged 5 – 11 years who weren’t yet eligible to be vaccinated at the time of interview, since the results from the vaccine trial had not been published.

Others were waiting for Novavax:

*We have talked about it and we've all, as a family, decided to wait for the Novavax one, which is a more traditional vaccine. It's one that we're all used to and it's not this mRNA thing that makes spike proteins. I've just heard so much bad stuff about it that I cannot go down that road. And we have discussed it, and they're all willing to wait as well. What's the big rush to get this thing in the arm? So Novavax apparently will be here in early February [2022]* *, and we’ll take it from there* - Messi

Finally, some parents were intending to indefinitely ‘refuse’ the vaccine for their children. All these parents had also refused the vaccine for themselves (Tables 2 and 3). They were not so concerned with short-term side effects, but were worried about the long-term, such as potential impacts on their child’s fertility. They also feared the “*unknown”* due to the vaccine being new:

*I'm nervous because it's an mRNA vaccine, which we haven't used before. We don't know what it's gonna do to our DNA long term. Because these are little people, like, their immune systems are pretty busy, they're not like completely developed yet, and we're just putting them into complete overdrive* – Anne

*I’d say that’s the biggest thing… I can't put something in their bodies that … I don't know how it's gonna affect them. There could also be adverse reactions….It's really just about their safety. How can you insist someone puts something on trial, provisionally approved, in their body that doesn't have long term research, how is that … it's not legal* – Eden

### Vaccine clinic preferences

For those who intended to vaccinate their children at some point, most wanted this to happen at a general practice (GP) clinic, with convenience and familiarity frequently reported as informing this preference. Participants regarded the immunisation providers there as being “*very experienced with administering immunisations all day long*.” They were also more comfortable with the GP being familiar to the child and having access to the child’s medical history/records. Some also noted “*it’s easy, [it’s] just down the road.”*

Parents’ views on school-based vaccination programs were mixed, regardless of whether their child was in primary or high school:

*Because our kids are at school from about 7.30 to 6 o’clock most days of the week… school would probably be our best* – Sebastian

*I probably wouldn’t want my children at a young age to get that vaccination at school… I would rather be there for them…my child wouldn’t want to get that done without a parent there, holding their hand* - Claire

Adele said she’d prefer for the first dose to be given by their doctor, but if it became an annual or routine practice, then she would be more accepting of a school-based program.

Few parents welcomed the idea of their children receiving their COVID-19 vaccines at a mass vaccination clinic, as these spaces felt unfamiliar and were not as conveniently located as a GP clinic. Some parents felt that mass clinics would potentially be “*overwhelming for some kids.”*

Finally, two parents said they’d vaccinate their children at a pharmacy due to the conveniency.

### COVID-19 vaccine decision-making

While participants often said that the decision to vaccinate was a discussion between themselves and their partner, many were acutely aware that they were making an important decision on behalf of their child. Because of this, some parents felt differently about COVID-19 vaccination of themselves compared to their children:

*I’m fully able to give consent and I can a hundred percent understand all the risks…my kids can't. So, it’s a decision I have to make for someone else, which makes me think slightly differently about it, maybe a bit more critically about it* - Nicholas

Often, parents of adolescents included them in the decision-making process:

*We tend to take a breadcrumbs approach sometimes with our kids, we sort of just suggest that they need to look into things and then we’re absolutely thrilled when we see the evidence that they've done it….When it was a possibility for my [15-year-old] daughter [to get vaccinated], we asked her if she would like to get vaccinated and if she had any questions or concerns about it, and she said “No, I want it and I want it as soon as I can get it.” So pretty much as soon as we could book her in, I think it might’ve been the day or the day after we could book a vaccination for her at one of the hubs, we booked in … and we took her to a vaccination hub and she’s had her first* – Pippin

Some described struggling with trying to respect their child’s autonomy over their health, but also wanting to protect them against COVID-19. Samuel said:

*My 13-year-old is often a difficult person to interact with at times and so it’s a strategic approach: the right time, the right place, the right influences. It’s not something that can be taken head on…. We won't force her to do so… If it's my decision, it would be yes [to her being vaccinated], but I need to take her on a journey. I'm not going to physically require her to get a vaccine. But there will be consequences from her decision making, it's not like it's just a free decision and there's no consequences. The consequences can be…some of the people that she's accustomed to interacting with, she may not be able to interact with anymore. [This] I do have influence over!*

When Lisa, who had no intention of vaccinating her 16-year-old daughter, was asked how she would feel if her daughter decided to get vaccinated, she said:

*That's her call … She's 16, she's a very mature young lady, and if that's what she feels she needs to do, that's her choice. I mean, same as the underage sex thing, you know, I’m not gonna be happy honey, but do it safely, you know, all that stuff. You know, same with smoking, I’m not gonna be bloody happy but if that's how you feel you've got to go with life, your choice. You know, I’m not gonna condemn her and kick her out.*

Some parents were comfortable in making the decision for their child following their own experiences:

*We would get our [children] vaccinated, for sure. I think particularly because by then hopefully my husband and I will be both fully vaccinated. I don't think I’d do if it was the other way around, if it was like, yeah, get your kids vaccinated and then not us. You know, like I think having the ability to be able to have the experience [myself] that it’s okay, I think that would give me more confidence to be able to then get the kids done –* Louise

However, Diana was of the opposite view, based on a desire to offer her child the strongest protection she could:

*I would have given her my vaccinations before me, no question. I'm much more afraid for my daughter getting sick, and she's got a much longer life ahead of her and of course I’d jump in front of a bus for her. So, yeah, she should come first, in the way I'd like it to be.*

Some parents seemed particularly eager to include their children in the decision making so that, should any long-term side effects occur, the parent would not be entirely to blame. Julia, as previously described, was prioritising other important life discussions with her adolescent son, and reflected on her desire to give him agency in the matter:

*My only concern with the teenagers is… if there’s side effects twenty years down the track, I don't want them to just go around and say, “Hey, you forced me to have this, and it's your fault that this happened to me.”*

### Concern about COVID-19

Parents’ level of concern about COVID-19 infection was a motivating factor for their intention to vaccinate their children or not. For those with a heightened sense of concern, most intended to vaccinate (or had already vaccinated) their children. Brooke, whose 11-year-old son has a congenital heart disease, said:

*They always say the kids are probably more resilient, [but] with COVID but you just don't know, if they've got that underlying health issue, how it's going to impact them. So that's my main concern…I have a really fit little man who has the heart issue but is living life fairly normally. But he could get COVID and it could take him down significantly.*

Louise, who had previously lost a child to an infectious disease, said that her *‘nightmares are made up of things like a worldwide virus that comes in and kills people.’*

Parents also became more concerned about COVID-19 as new variants came into circulation in Australia’s Eastern states. For the parents with lower concerns about the disease, all but one (Ruby) did intend to vaccinate their child at some point. Their relaxed approach seemed to stem mostly from the fact that WA, at the time of data collection, had very controlled interstate and international borders to keep COVID-19 out:

*I think my concerns [are] lower because we live in WA. There's been no cases for a long time. I think if I was living in maybe Sydney or Melbourne…then yeah, I would have some concerns* - Jurgen

Very few parents had *no* concern about COVID, and they tended to be parents of older children (15 – 16 years). Anne was an exception. She was the stepmother of two 5-year-olds, and she believed scare tactics were being used to make certain populations concerned about COVID-19 coincidentally at the same time that the vaccine or booster doses were being recommended for those populations:

*Children haven't been affected by COVID until the vaccine has been approved. So suddenly children are getting COVID, which I find is very interesting how it's kind of coincided with the approval for 5- to 11-year-olds to have the vaccine.*

The parents with no concerns intended to either ‘wait awhile’ for the vaccine, or ‘refuse’ it. Messi, who was ‘waiting awhile’ for Novavax, was not afraid of COVID-19:

*We're fit and healthy, we're always doing the right thing, we eat well, eat plenty of vitamins. I'm not afraid of COVID at all, no, and I wouldn't be for my children or my wife.*

### Social network

Approximately half of our participants said that COVID-19 vaccination of children was not a topic of conversation for parents in their social network. They and their peers avoided talking about it because they regarded vaccination as “*too personal*” or potentially controversial to discuss with others. Also, for many, being able to vaccinate their children was not yet a reality and felt “so far away.” Marty, who was interviewed in September about his children aged < 12 years, said:

*I actually haven't had a lot of discussion with other parents about vaccination of children. I think maybe because largely it is the parents’ decision, and the fact that there isn't one available yet, that people aren’t talking about it so much. I know me and my wife do talk about it, but not really to other parents.*

However, the other half of our parents were speaking with their peers about COVID-19 vaccination. Conversations were extremely varied and some parents reported varying levels of vaccine support amongst their peers. Some described conversations about the benefits of vaccinating children and had heard from others about their positive experiences. Some had also discussed with peers about whether or not they’d let their adolescent children choose for themselves. Others had discussed the potential long-term side effects of the vaccine or general hesitancy. For example, both Eden and Brooke had experienced other parents expressing concern about the vaccine’s impact on children’s future fertility:

*I mean, people who I’ve heard who are hesitant to get their children done, they were like, “We just don't know how it's going to impact them potentially having children in a few years,” and I'm like, “It's hard, it is a hard decision.” And I was talking to hubby [husband], you know, just “do we do this?”…There's been so many other vaccines that we've not second guessed like this … things like the HPV one, the flu shot that we give the kids every year. You know, we don't second guess that in regards to their fertility, so why should we with this one?* - Brooke

*I've got a friend who's got a 9-year-old, this was my best friend. She said “I'm gonna have to get my daughter... her eggs frozen, I'm gonna get her eggs frozen, because I just don't know the long term*“- Eden

Regardless of whether or not participants had discussed COVID-19 vaccination with other parents, the majority told us that their peers’ behaviour would not influence them:

*Unless they’re a medical specialist who can advise me on a medical based background… I don't care what they think or do, other than their failure to vaccinate [which] could have implications on how much we interact with them* - Jim

### Information needs

We asked participants what strategies the government or health care providers could implement to help parents vaccinate their children against COVID-19. The only suggestion made was for an education campaign for children of all ages, as parents had not yet come across any information. Although this may be due to some of our interviews being conducted before younger children were eligible to be vaccinated, this was not the case for all. For example, Caroline’s son was able to be vaccinated but hadn’t yet been due to her concerns about short-term side effects. She said:

*I don't think there is a huge push, to be honest, like, for kids. It's more adults, and it's, like, essential workers, you know, where I just don't feel like there's any marketing… for kids.*

Milly, who works at a multicultural centre, described how a lack of official information is then filled in by misinformation:

*We have a lot of migrants from outside who don't speak English. So what happens is, whatever they’re told by their families and friends, they just believe that. So I guess there's a lot out there which is just there, and people end up believing it … [T]hey don't know what the source of the information is, you end up reading it and believing it and it causes some doubts in your mind. So I think there should be more for, like, alert, like bring it out in the open, talk more about it, more information about the vaccine, so people know about it more and then, you know, says that it's safe enough to get it done.*

Overwhelmingly, parents wanted information on vaccine safety above all else, such as “*confirmation that there’s minimal serious side effects*,” what to expect immediately after vaccination, possible longer-term effects, and when to seek medical attention:

*[I’d like] information that says it’s safe. I guess the percentage that do get reactions and adverse reactions. So, you know, I’d imagine it’d be very, very low number. And I think that would give me confidence reading that, okay that's very, very, very small risk* – Marty

*I think you have to be honest and real about it, so … what's in the normal range, what's outside a normal range and what do you need to seek medical attention for, so you're really clear on, okay, if this happens and it goes for this long, then that's outside a normal range and you need to seek help -* Tracey

Many regarded a public information campaign featuring facts on COVID-19 vaccine safety for children to be reassuring, rather than off-putting, as it would show that the government was being transparent and honest with both parents and children (who would potentially also see campaign materials), and also that many of the side effects are minor:

*For me I think it would be reassuring, but I know there’s a lot of people who take it out of context. You know, there's a lot of people who say there’s 10,000 people with adverse reactions to a COVID vax, whereas 9,800 of those are a sore shoulder, so I think it can be misconstrued* - Nicholas

*I think you've got to stop mamby-pambying everyone and be just straight to the point. … Be realistic, and I don't care if they're off putting! They need to know , and it's really no big deal, you know, if you have side [effects] … “I wasn't well for three days”… Better than being dead [from COVID-19]! I think they’ve got to stop tiptoeing around them and making them feel so safe all the time. Children, they need to be a bit more resilient … I think just hit them with the hard facts* – Sal

The other main topic parents were eager to see in a campaign was on the benefit of vaccinating children for both the children themselves, but also the broader community:

*Benefits…I think parents go a lot on benefits… to children…why is [vaccination] good for them…that's reassuring. I think parents in general need to be reassured* – Julia

*Understanding the benefits, not just for your child or for the community, or highlighting the benefits of when most people are vaccinated, how we can open up [our borders] and get back to normal* - Marty

Further to featuring information on side effects, which would show transparency from the government, participants were also eager for clear messaging. Some felt that the communication at the time downplayed the potential risk of COVID-19 to children, which then decreased motivation to vaccinate:

*I think that them [experts] saying that children don't get COVID, and particularly that message through social media, will make it incredibly hard to get people publicly to be able to immunise their children* – Louise

*I think just making sure the messaging is really clear, [and] being very methodical about it … We've had different stuff come from Commonwealth and … obviously to date the [vaccine] rollout hasn't been handled probably as good as it could’ve been. So, I think if they can kind of improve in that space and just have consistent messaging from a single source that's accurate and up-to-date just so you know where to go if you have queries or you've got any questions -* Tracey

Participants said campaign information could either be strictly factual, or feature personal stories from parents – doing both would cater to a wider range of parents. They wanted to see information shared through social media, GP clinics, emails from the government, television, YouTube, schools, playgroups, or regularly updated fact sheets or websites in plain language (but including references for further reading):

*When governments are putting out, like, a PDF or a website with fairly generic and simple language, the ability to drill down to supporting research I think would be really advantageous. Not saying that people will actually follow, people like to read the headline and maybe pass it on before they actually read the content. But having more information there that goes down to I guess … we're not gonna cascade down to, like, a medical paper, necessarily, but having those available for people who did want to access it I think would add to credibility -* Samuel

## Discussion

This qualitative study provided an understanding of how West Australian parents were thinking and feeling about COVID-19 and COVID-19 vaccination of their school-aged children at the time they were becoming eligible for vaccination. As it happened more broadly in Australia, there was a global disconnect in uptake between children and adolescents, in which uptake was higher among adolescents [41, 42]. Our study may offer some insights, including the sequential roll out of the vaccine, the impact of policies, the involvement of adolescents in the decision making, and parents’ understanding of disease severity. Overall, however, our findings need to be considered with the understanding that many parts of Australia, including WA, were effectively COVID-free for the first two years of the pandemic due to hard border closures and other non-pharmaceutical interventions. This led some of our participants in this and other studies feel they could “wait awhile” and vaccinate when they felt at greater threat of the disease [38]. With the arrival of the SARS-CoV2 Omicron variant in November 2021 and the subsequent opening up of international and state borders, COVID-19 epidemiology changed dramatically in Australia, with extensive community transmission in all jurisdictions.

While much research on childhood vaccination focuses on vaccine hesitancy for routine National Immunisation Program (NIP) vaccines, it is important to note that the participants in this sample were all supportive of these vaccinations and the vast majority were fully vaccinated (according to parental report); many were also very accepting of the influenza vaccine. The one child not up-to-date with their vaccines was unvaccinated on medical advice. However, despite parents mostly being on the same page about NIP vaccines, we found significant variability in their attitudes towards COVID-19 vaccines for their children in late 2021. However, as 2022 COVID-19 vaccination rates in those 12–15 year old began to resemble uptake of routine adolescent vaccines (three dose Human Papillomavirus Vaccine Coverage, 2017; 80% for females; 76% for males), [43] there was indication that Australian parents began to consider COVID-19 vaccination of equivalent importance as other NIP vaccines.

Parents in our study overwhelmingly told us that what their social network was doing would not impact the decisions they made. However, about half of them did report considerable ‘chatter’ with peers on the issue. With previous research on childhood vaccination indicating that the vaccination beliefs of peers in one’s social network is the strongest predictor of one’s vaccination status, there is likely a disconnect between what people *think* is the effect of peer influence and what its effects actually are [44]. Studies conducted in tight-knit communities where vaccine refusal is prevalent have found that the behaviour and attitudes of others can be highly influential on not only what parents do about vaccination, but also on whether they reveal that they’ve vaccinated their own children, with impacts for the prevailing social norms [45–47]. A similar result was found in our team’s work on young adults, [48] where participants stated they would not be influenced to change their decision about receiving a vaccine even if uptake those in their social network decided differently.

When applying our model of vaccine intentions developed for adults making decisions about their own COVID-19 vaccination, [38] we found a conflict between what parents might be wanting for their children in terms of vaccination and parents’ intentions for respecting their adolescent children’s autonomy over their own health. Sometimes this generated conflictual positions, with some adolescents making their own decisions regardless of what their parents wanted. The dynamic of decision-making and negotiation between parents and their older children regarding vaccination has been explored previously in global research on the HPV vaccine, with mixed results regarding who makes the decision. A US qualitative study looking at the vaccination of boys found that both parents and sons felt the child had a role in decision making and it was generally their first time being included in medical decisions [49]. Another qualitative study found that parents were the primary decision makers, but as children got older they had more input; however some parents expressed leaving the decision up to their child [50]. Quantitative studies generally found mothers were primary decision maker, [51] but felt adolescents had a say in decision making [52, 53].

With regard to fear and misinformation, our participant Milly described how misinformation can get into culturally and linguistically diverse communities, as we explored in a separate study [54]. But in *any* community, gaps in public information and knowledge can be filled by misinformation. While it’s hard (or impossible) for governments and health care providers to share detailed safety information before the vaccine trial results are published or before technical authorities have approved the vaccine, perhaps the time leading up to this could have been better used to describe the benefit of vaccinating children once the vaccine became available. Instead, in Australia, medical and health experts sought to reassure parents that their children would be likely to experience COVID as a mild illness and to make a full recovery. As our participants Louise and (more conspiratorially) Anne observed, this then required a rather sudden about-face once vaccines were available for adolescents and children, and authorities wanted parents to accept them.

We also recommend that authorities share more information and resources regarding what goes into a vaccine trial in order for the scientists concerned to learn that the vaccine is safe. It may be fruitful to share additional information about post-vaccination safety surveillance systems and to reassure parents that health systems continue to track vaccines’ safety well after their administration. Filling the communication space with this information may be a way of preventing its colonisation by misinformation. These lessons will be important for future pandemics.

While our study captured important insights at the beginning of a rollout of a new vaccine for children and adolescents, it is not without limitations. Firstly, it is not generalisable to the entire paediatric/adolescent cohort in Australia: this is because of the qualitative methods we employed, but also given the unique context in which we collected data whereby WA had yet to feel the impact of widespread COVID-19 transmission throughout the state. Secondly, many of our participants were highly educated and lived within the Perth metropolitan area, and thus we cannot be sure that our results reflect what parents in rural WA think and feel about COVID-19 vaccination for their children, nor what those with lower health literacy understand about vaccination. This may be a result of our passive recruitment efforts through mainstream and social media, which required potential participants to then be computer literate to sign up on the REDCap survey and be able to understand the English language. Further, while we sought to capture the voice of parents of children with comorbidities that increase the risk of a severe COVID-19 infection, we faced challenges recruiting this group and they were only a minority in the final cohort once we had undertaken a clinical review of the self-reported conditions (four parents who signed up as having children with comorbidities did not actually have comorbidities that increase the risk of a severe COVID-19 infection). Finally, our results may not reflect the experiences of parents of Aboriginal children in Perth – we are undertaking a separate project through Coronavax and working with the Community to understand these experiences.

## Conclusion

While many parents of school-aged children in Australia ultimately vaccinated their children against COVID-19, some parents, even those who usually accept the National Immunisation Program and influenza vaccines for their children, did not. Our study offers insight into some potential reasons for refusal, including concerns about mRNA technology, impacts on fertility, low disease risk perception, awaiting a recommendation from a familiar health care provider, and the perception that there has been little to no information/campaigning directed towards parents to help them, and their children, with their decision making. For future pandemic vaccination initiatives extending to children it will be important for governments, health professionals and official messengers to address parents’ concerns, as well as their vaccine preferences, when developing strategies to assist families in understanding and accessing vaccination.

## Data Availability

The datasets generated and/or analysed during the current study are not publicly available due to the constraints of ethical approval but are available from the corresponding author on reasonable request.

## Abbreviations

COVID-19: Coronavirus Disease 2019
MIS-C: Multisystem Inflammatory Disease in Children
NIP: National Immunisation Program
PIMS-TS: Paediatric Inflammatory Multisystem Syndrome
US: United States
WA: Western Australia

## References

[1] COVID-19 National Incident Room Surveillance Team (2021). COVID-19 Australia: epidemiology report 55. Commun Dis Intell.

[2] Hobbs CV, Woodworth K, Young CC, Jackson AM, Newhams MM, Dapul H (2022). Frequency, characteristics and complications of COVID-19 in hospitalized infants. Pediatr Infect Dis J.

[3] Mukkada S, Bhakta N, Chantada GL, Chen Y, Vedaraju Y, Faughnan L (2021). Global characteristics and outcomes of SARS-CoV-2 infection in children and adolescents with cancer (GRCCC): a cohort study. Lancet Oncol.

[4] Williams P, Koirala A, Saravanos G, Lopez L, Glover C, Sharma K (2022). COVID-19 in New South Wales children during 2021: severity and clinical spectrum. Med J Aust.

[5] Zimmermann P, Pittet LF, Curtis N (2021). How common is long COVID in children and adolescents?. Pediatr Infect Dis J.

[6] WA Department of Education, WA Department of Health, Telethon Kids Institute. DETECT schools study: understanding the impact of COVID-19 in Western Australian schools. 2021. https://www.telethonkids.org.au/globalassets/media/documents/projects/detect-schools-study-final-report-and-appendices.pdf. Accessed 30 Mar 2022.

[7] Racine N, McArthur BA, Cooke JE, Eirich R, Zhu J, Madigan S (2021). Global prevalence of depressive and anxiety symptoms in children and adolescents during COVID-19: a meta-analysis. JAMA Pediatr.

[8] Australian Technical Advisory Group on Immunisation (ATAGI. ATAGI statement on the use of COVID-19 vaccines in all young adolescents in Australia. 2021. https://www.health.gov.au/news/atagi-statement-on-the-use-of-covid-19-vaccines-in-all-young-adolescents-in-australia. Accessed 29 Apr 2022.

[9] Australian Technical Advisory Group on Immunisation (ATAGI). ATAGI recommendations on the use of the paediatric Pfizer COVID-19 vaccine in children aged 5 to 11 years in Australia. 2022. https://www.health.gov.au/resources/publications/atagi-recommendations-on-pfizer-covid-19-vaccine-use-in-children-aged-5-to-11-years. Accessed 29 Apr 2022.

[10] WA Department of Health. COVID-19 vaccines for children aged 6 months to under 5 years. 2022. https://www.health.gov.au/initiatives-and-programs/covid-19-vaccines/who-can-get-vaccinated/covid-19-vaccines-for-children-aged-6-months-to-under-5-years. Accessed 6 Sep 2022.

[11] National Centre for Immunisation Research and Surveillance. Significant events in COVID-19 vaccination practice in Australia. 2022. https://www.ncirs.org.au/sites/default/files/2022-02/COVID-19-history-February%202022_0.pdf. Accessed 30 Mar 2022.

[12] McGowan M, Sanderson A-J. COVID-19 vaccination bookings open for five to 11-year-olds. 2021. https://www.mediastatements.wa.gov.au/Pages/McGowan/2021/12/COVID-19-vaccination-bookings-open-for-five-to-11-year-olds.aspx. Accessed 30 Mar 2022.

[13] McGowan M, Cook R, Ellery S: WA parents called on to support COVID-19 teen jab effort. 2021. https://www.mediastatements.wa.gov.au/Pages/McGowan/2021/09/WA-parents-called-on-to-support-COVID-19-teen-jab-effort.aspx. Accessed 30 Mar 2022.

[14] Operation COVID Shield. COVID-19 vaccine rollout as of 22 August 2022. 2022. https://www.health.gov.au/sites/default/files/documents/2022/08/covid-19-vaccine-rollout-update-22-august-2022.pdf. Accessed Sep 2022.

[15] Casiday R, Cresswell T, Wilson D, Panter-Brick C (2006). A survey of U.K. parental attitudes to the MMR vaccine and trust in medical authority. Vaccine..

[16] Harmsen IA, Mollema L, Ruiter RAC, Paulussen TGW, de Melker HE, Kok G (2013). Why parents refuse childhood vaccination: a qualitative study using online focus groups. BMC Public Health.

[17] Fournet N, Mollema L, Ruijs WL, Harmsen IA, Keck F, Durand JY (2018). Under-vaccinated groups in Europe and their beliefs, attitudes and reasons for non-vaccination; two systematic reviews. BMC Public Health.

[18] Gross K, Hartmann K, Zemp E, Merten S (2015). 'I know it has worked for millions of years': The role of the 'natural' in parental reasoning against child immunization in a qualitative study in Switzerland. BMC Public Health.

[19] Attwell K, Leask J, Meyer SB, Rokkas P, Ward PR (2017). Vaccine rejecting parents' engagement with expert systems that inform vaccination programs. J Bioeth Inq.

[20] Ward PR, Attwell K, Meyer SB, Rokkas PR, Leask J (2017). Understanding the perceived logic of care by vaccine-hesitant and vaccine-refusing parents: A qualitative study in Australia. PLoS ONE.

[21] Reich JA (2014). Neoliberal mothering and vaccine refusal: imagined gated communities and the privilege of choice. Gend Soc.

[22] Leask J, Chapman S, Hawe P, Burgess M (2006). What maintains parental support for vaccination when challenged by anti-vaccination messages? A qualitative study. Vaccine.

[23] Enkel SL, Attwell K, Snelling TL, Christian HE (2018). ‘Hesitant compliers’: Qualitative analysis of concerned fully-vaccinating parents. Vaccine.

[24] Bell S, Clarke R, Mounier-Jack S, Walker JL, Paterson P (2020). Parents’ and guardians’ views on the acceptability of a future COVID-19 vaccine: A multi-methods study in England. Vaccine.

[25] Walker KK, Head KJ, Owens H, Zimet GD (2021). A qualitative study exploring the relationship between mothers’ vaccine hesitancy and health beliefs with COVID-19 vaccination intention and prevention during the early pandemic months. Hum Vaccin Immunother.

[26] Yoda T, Katsuyama H (2021). Parents’ hesitation about getting their children vaccinated against COVID-19 in Japan. Hum Vaccin Immunother.

[27] Goldman RD, Bone JN, Gelernter R, Krupik D, Ali S, Mater A (2021). National COVID-19 vaccine program progress and parents’ willingness to vaccinate their children. Hum Vaccin Immunother.

[28] Bianco A, Della Polla G, Angelillo S, Pelullo CP, Licata F, Angelillo IF (2022). Parental COVID-19 vaccine hesitancy: a cross-sectional survey in Italy. Expert Rev Vaccines.

[29] Goldman RD, Ceballo R, The International Covid-Parental Attitude Study Group. (2022). Parental gender differences in attitudes and willingness to vaccinate against COVID-19. J Paediatr Child Health..

[30] Ruggiero KM, Wong J, Sweeney CF, Avola A, Auger A, Macaluso M (2021). Parents’ intentions to vaccinate their children against COVID-19. J Pediatr Health Care.

[31] Goldman R, Yan T, Seiler M, Parra Cotanda C, Brown J, Klein E (2020). Caregiver willingness to vaccinate their children against COVID-19: Cross sectional survey. Vaccine.

[32] Bagateli LE, Saeki EY, Fadda M, Agostoni C, Marchisio P, Milani GP (2021). COVID-19 vaccine hesitancy among parents of children and adolescents living in Brazil. Vaccines.

[33] Evans S, Klas A, Mikocka-Walus A, German B, Rogers G, Ling M (2021). “Poison” or “protection”? A mixed methods exploration of Australian parents' COVID-19 vaccination intentions. J Psychosom Res.

[34] Government of Western Australia: Department of Health. Coronavirus COVID-19 in Western Australia. 2022. https://experience.arcgis.com/experience/359bca83a1264e3fb8d3b6f0a028d768. Accessed Apr 29 2022.

[35] Attwell K, Carlson S, Tchilingirian J, Harper T, McKenzie L, Roberts L (2021). Coronavax: preparing community and government for COVID-19 vaccination: a research protocol for a mixed methods social research project. BMJ Open.

[36] Harris PA, Taylor R, Minor BL, Elliott V, Fernandez M, O'Neal L (2019). The REDCap consortium: Building an international community of software platform partners. J Biomed Inform.

[37] Harris PA, Taylor R, Thielke R, Payne J, Gonzalez N, Conde JG (2009). Research electronic data capture (REDCap)—a metadata-driven methodology and workflow process for providing translational research informatics support. J Biomed Inform.

[38] Carlson SJ, McKenzie L, Roberts L, Blyth CC, Attwell K (2022). Does a major change to a COVID-19 vaccine program alter vaccine intention? A qualitative investigation. Vaccine.

[39] Attwell K, Keays E, McKenzie L, Roberts L, Blyth CC, Carlson SJ (2023). Mandating COVID-19 vaccinations for children: Attitudes of Western Australian parents. Aust J Soc Issues.

[40] Australian Bureau of Statistics. 2033.0.55.001 Socio-Economic Indexes for Australia (SEIFA), 2016. 2016. https://www.abs.gov.au/AUSSTATS/abs@.nsf/DetailsPage/2033.0.55.0012016. Accessed 2 Jul 2021.

[41] American Academy of Pediatrics. Children and COVID-19 vaccination trends. 2022. https://www.aap.org/en/pages/2019-novel-coronavirus-covid-19-infections/children-and-covid-19-vaccination-trends. Accessed 20 Sep 2022.

[42] National Health Service. COVID-19 vaccination statistics: week ending Sunday 4th September 2022. 2022. https://www.england.nhs.uk/statistics/wp-content/uploads/sites/2/2022/09/COVID-19-weekly-announced-vaccinations-08-September-2022.pdf. Accessed 20 Sep 2022.

[43] Department of Health. National HPV 3 dose vaccination coverage for all adolescents turning 15 years of age from year of program commencement. 2020. https://www.health.gov.au/resources/publications/national-hpv-3-dose-vaccination-coverage-for-all-adolescents-turning-15-years-of-age-from-year-of-program-commencement. Accessed 29 Apr 2022.

[44] Brunson EK (2013). The impact of social networks on parents’ vaccination decisions. Peds.

[45] Sobo EJ (2015). Social cultivation of vaccine refusal and delay among Waldorf (Steiner) school parents. Med Anthropol Q.

[46] Attwell K (2019). The politics of picking: Selective vaccinators and population-level policy. SSM Popul Health.

[47] Attwell K, Meyer S, Ward P (2018). The social basis of vaccine questioning and refusal: A qualitative study employing Bourdieu’s concepts of ‘capitals’ and ‘habitus’. Int J Environ Res Public Health.

[48] Roberts L, McKenzie L, Carlson S, Tomkinson S, Attwell K (2023). Reopening to the world: How safety, normality and trust in government shape young adults’ COVID-19 vaccine intentions. Aust J Polit Sci.

[49] Alexander AB, Stupiansky NW, Ott MA, Herbenick D, Reece M, Zimet GD (2012). Parent-son decision-making about human papillomavirus vaccination: a qualitative analysis. BMC Pediatr.

[50] Gowda C, Schaffer SE, Dombkowski KJ, Dempsey AF (2012). Understanding attitudes toward adolescent vaccination and the decision-making dynamic among adolescents, parents and providers. BMC Public Health.

[51] Berenson AB, Laz TH, Hirth JM, McGrath CJ, Rahman M (2014). Effect of the decision-making process in the family on HPV vaccination rates among adolescents 9–17 years of age. Hum Vaccin Immunother.

[52] Herman R, McNutt L-A, Mehta M, Salmon DA, Bednarczyk RA, Shaw J (2019). Vaccination perspectives among adolescents and their desired role in the decision-making process. Hum Vaccin Immunother.

[53] La Vincente S, Mielnik D, Jenkins K, Bingwor F, Volavola L, Marshall H (2015). Implementation of a national school-based Human Papillomavirus (HPV) vaccine campaign in Fiji: knowledge, vaccine acceptability and information needs of parents. BMC Public Health.

[54] Carlson SJ, Edwards G, Blyth CC, Nattabi B, Attwell K (2022). Corona is coming”: COVID-19 vaccination perspectives and experiences amongst Culturally and Linguistically Diverse West Australians. Health Expect.

